# Nitrogen fertilization of intercropped cereal-legume: A potassium, sulfur, magnesium and calcium plant acquisition dataset

**DOI:** 10.1016/j.dib.2022.107816

**Published:** 2022-01-10

**Authors:** Edith Le Cadre, Ana Beatriz de Oliveira, Mustapha Arkoun, Jean Claude Yvin, Philippe Hinsinger

**Affiliations:** a SAS, INRAE, Institut Agro, Rennes 35042, France; b Centre Mondial de l'Innovation, Saint Malo, France; c INRAE, CIRAD, IRD, University Montpellier, Institut Agro, Montpellier, France

**Keywords:** Plant mixtures, Fertilization, Gradient, Competition, Nutrient, Abiotic stress

## Abstract

Cereal-legume mixture is a well-known successful intercrop model for an efficient use of soil nutrients [1,2]. Effects of mineral N gradient on the acquisition of major nutrients: potassium (K), calcium (Ca), magnesium (Mg) and sulfur (S) is presented. A greenhouse pot experiment was conducted with wheat (Triticum aestivum L. cv. Lennox) and white lupin (Lupinus albus L. cv. Feodora) grown as sole crops and intercropped along a soil mineral N gradient obtained by ^15^N addition. Plants were harvested at flowering stage and dry weights of shoots and roots were measured. Potassium, calcium, magnesium and sulfur concentrations in shoots and roots were determined by Inductively Coupled Plasma Mass Spectrometry (ICP-MS).

**Specifications Table**

**Table:** 

Subject	Agricultural sciences
Specific subject area	Agronomy and crop sciences
Type of data	Table figure
How data were acquired	Dataset was acquired after a greenhouse experiment and by using Inductively Coupled Plasma Mass Spectrometry for plant tissue analysis
Data format	Raw data Analysed data
Parameters for data collection	Mediterranean soil
Description of data collection	Data collection was acquired in two step: Step 1. A greenhouse pot experiment during 61 days, using a full factorial design (7 replicates) with three N treatments (N1 = 2, N2 = 33 and N3 = 65 mg N kg^−1^ dry soil added as a ^15^N-labelled urea) and three crop treatments: wheat (Triticum aestivum L. cv. Lennox) grown as sole crop; white lupin (Lupinus albus L. cv. Feodora) grown as sole crop. Step 2. The dry weights of shoots and roots were measured after drying at 70 °C for 3 days. Potassium, Ca, Mg and S concentrations in shoots and roots were determined by Inductively Coupled Plasma Mass Spectrometry (ICP-MS) after digestion of 100 mg of finely ground material in a microwave oven (Multiwave PRO, Anton Paar, Austria) with concentrated HNO_3_ (65%) at 200 °C.
Data source location	Institution: Institut Agro City/Town/Region: Rennes Country: France Latitude and longitude for collected samples/data: Mauguio in Southern France (3°59′6″E, 43°37′13″N)
Data accessibility	Repository: https://data.inrae.fr/privateurl.xhtml?token=af39875b-4591–4e34-af77-beea6dfb02b5 doi:10.15454/8PNFDE

**Value of the Data**

• Intercropping is an agroecological solution emerging as an alternative to dominant systems requiring high inputs of nutrients.
• This dataset can be used to identify critical N dose shifting plant interactions.
• This dataset can be used to design field trials to phenotype plants for their ability to coexist under resource gradient.
• Plant breeders and cropping systems designers are the panel targeted by this dataset in order to identify multi-nutrient acquisition trait trade-offs in intercropped systems.

## 1 Data Description

### 1.1 Differences of nutrient accumulation of mono and intercropped species along nitrogen gradient

**Fig. 1:**
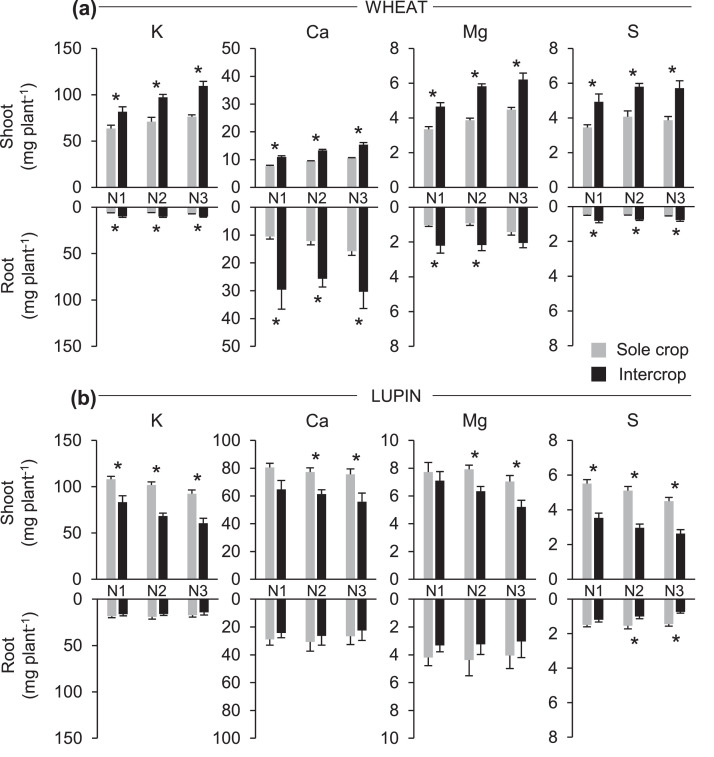
Potassium (K), calcium (Ca), magnesium (Mg) and sulfur (S) accumulation (mg plant^−1^) in shoots (upper bars) and roots (lower bars) of wheat (a) and lupin (b) as sole crops or as intercrops within each N treatment (N1, N2, N3). Values are the mean of 7 replicates. Error bars represent standard errors. Stars stand for significant differences between crop treatments within each N treatment (Tukey's test at *p* < 0.05).

**Fig. 2:**
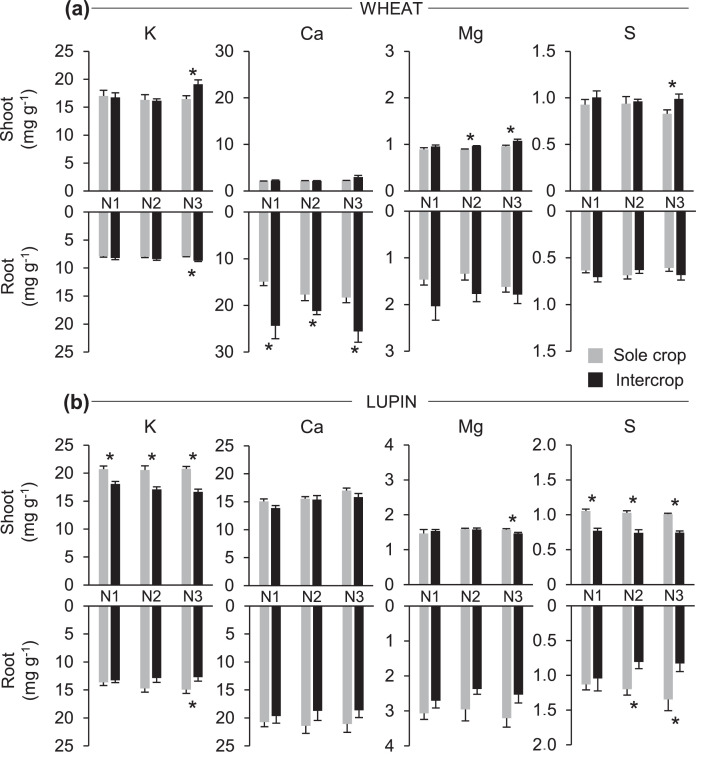
Potassium (K), calcium (Ca), magnesium (Mg) and sulfur (S) concentrations (mg g^−1^ of dry weight) in shoots (upper bars) and roots (lower bars) of wheat (a) and lupin (b) as sole crops or as intercrops within each N treatment (N1, N2, N3). Values are the mean of 7 replicates. Error bars represent standard errors. Stars stand for significant differences between crop treatments within each N treatment (Tukey's test at *p* < 0.05).

**Fig. 3:**
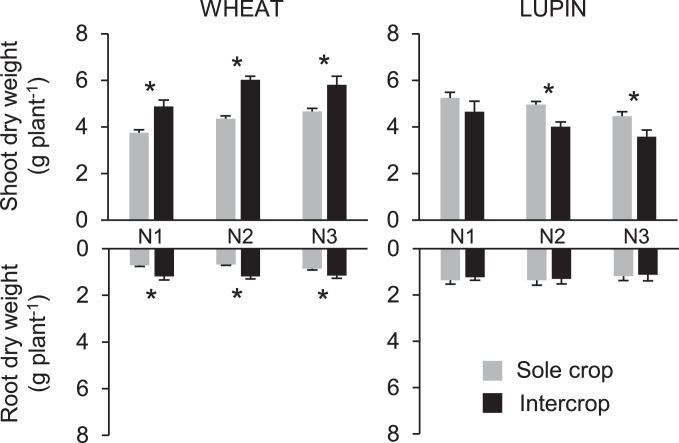
Dry weight of shoots and roots of wheat and lupin as sole crops or as intercrops within each N treatment (N1, N2, N3). Values are the mean of 7 replicates. Error bars represent standard errors. Stars stand for significant differences between crop treatments within each N treatment (Tukey's test at *p* < 0.05).

Within each soil N treatment (Table 3), intercropping systematically induced a positive effect on wheat and a negative effect on lupin, whether in terms of nutrient concentration or accumulation (Figs. 1 and 2; Table 1). Except for Mg in roots at N3, intercropped wheat systematically and significantly accumulated more K, Ca, Mg and S in both shoots and roots, whatever the N treatment (Fig. 1a, Tables 2 and 3) compared to sole wheat. In contrast, intercropped lupin exhibited lower significant nutrient accumulation observed for shoots (Fig. 1b, Tables 2 and 3) compared to sole lupin. Except for Ca and Mg at N1, a significant decrease of K, Ca, Mg and S accumulation in shoots was observed for intercropped lupin compared to sole lupin, whatever the N treatment. In particular, sulphur accumulation were lower for intercropped S shoots whatever N treatment (Fig. 1a and Table 3) whereas lupin intercropped roots were only affected at N2 and N3 compared to sole crop. In contrast, S accumulations in intercropped wheat were systematically greater than those of sole wheat at all N levels (Fig. 1a, Table 3). These results were consistent with the greater dry weight of shoots and roots of intercropped wheat is observed compared to sole wheat, whatever the N treatment, and lower shoot dry weight of intercropped lupin at N2 and N3 (Fig. 3) compared to sole lupin.

**Table 1:** Results (*p*-values thresholds) of two-way ANOVA (crop treatment x N treatment) performed on potassium (K), calcium (Ca), magnesium (Mg) and sulfur (S) accumulation in shoots and roots of wheat and lupin as sole crops or as intercrops.

	SHOOT							
	K		Ca		Mg		S	
	Wheat	Lupin	Wheat	Lupin	Wheat	Lupin	Wheat	Lupin
Crop treatment	< 0.01	< 0.001	< 0.001	< 0.05	< 0.001	ns	< 0.01	< 0.001
N treatment	ns	ns	< 0.001	ns	< 0.01	ns	ns	< 0.05
Crop treatment x N treatment	ns	ns	ns	ns	ns	ns	ns	ns

	ROOT							
	K		Ca		Mg		S	
	Wheat	Lupin	Wheat	Lupin	Wheat	Lupin	Wheat	Lupin

Crop treatment	< 0.001	ns	< 0.01	ns	< 0.01	ns	< 0.001	ns
N treatment	ns	ns	ns	ns	ns	ns	ns	ns
Crop treatment x N treatment	ns	ns	ns	ns	ns	ns	ns	ns

**Table 2:** Results (*p*-values thresholds) of two-way ANOVA (crop treatment x N treatment) performed on potassium (K), calcium (Ca), magnesium (Mg) and sulfur (S) concentration in shoots and roots of wheat and lupin as sole crops or as intercrops.

	SHOOT							
	K		Ca		Mg		S	
	Wheat	Lupin	Wheat	Lupin	Wheat	Lupin	Wheat	Lupin
Crop treatment	ns	< 0.001	ns	< 0.05	< 0.001	ns	< 0.05	< 0.001
N treatment	ns	ns	ns	< 0.01	< 0.001	ns	ns	ns
Crop treatment x N treatment	ns	ns	ns	ns	ns	ns	ns	ns

	ROOT							
	K		Ca		Mg		S	
	Wheat	Lupin	Wheat	Lupin	Wheat	Lupin	Wheat	Lupin

Crop treatment	< 0.01	< 0.01	< 0.001	<0.05	< 0.05	< 0.01	ns	< 0.001
N treatment	ns	ns	ns	ns	ns	ns	ns	ns
Crop treatment x N treatment	ns	ns	ns	ns	ns	ns	ns	ns

**Table 3:** Results (*p*-values) of one-way ANOVA (crop treatment as factor meaning intercropped or sole crop) performed on potassium (K), calcium (Ca), magnesium (Mg) and sulfur (S) accumulation or concentration in shoots and roots of wheat and lupin as sole crops or as intercrops within each N treatment (N1, N2, N3). Number of stars (one, two or three) indicates the test significance level (*p* < 0.05, *p* < 0.01 or *p* < 0.001, respectively).

	WHEAT				LUPIN			
N	Accumulation in Shoots				Accumulation in Shoots			
treatments	K	Ca	Mg	S	K	Ca	Mg	S
N1	0.0155*	0.0001***	0.0005***	0.0104*	0.0067**	0.0603	0.5156	0.0001***
N2	0.0005***	0.0000***	0.0000***	0.0007***	0.0000***	0.0030**	0.0056**	0.0000***
N3	0.0000***	0.0000***	0.0008***	0.0022**	0.0005***	0.0167*	0.0155*	0.0001***

	Accumulation in Roots				Accumulation in Roots			
	K	Ca	Mg	S	K	Ca	Mg	S

N1	0.0051**	0.0134*	0.014*	0.006**	0.4238	0.4207	0.2601	0.1012
N2	0.0002***	0.0012**	0.003**	0.0005***	0.2706	0.6582	0.4204	0.0289*
N3	0.0063**	0.0379*	0.0805	0.0043**	0.406	0.6687	0.5083	0.0004***

	Concentration in Shoots				Concentration in Shoots			
	K	Ca	Mg	S	K	Ca	Mg	S

N1	0.8376	0.2246	0.1734	0.4021	0.0024**	0.0891	0.5795	0.0000***
N2	0.8984	0.7729	0.0004***	0.7582	0.0017**	0.8375	0.8536	0.0002***
N3	0.0183*	0.0808	0.0186*	0.0341*	0.0000***	0.1625	0.0279*	0.0000***

	Concentration in Roots				Concentration in Roots			
	K	Ca	Mg	S	K	Ca	Mg	S

N1	0.3414	0.0071**	0.1010	0.2144	0.6050	0.4989	0.2083	0.6677
N2	0.1674	0.0386*	0.0667	0.3359	0.0951	0.2421	0.1335	0.0091**
N3	0.0024**	0.0165*	0.4724	0.2552	0.0417*	0.2371	0.0776	0.0241*

### 1.2 Plant tissue concentration

The only significant differences between crop treatments for K concentrations in wheat were observed at N3 (Fig. 2a, Tables 2 and 3). In contrast, for all N treatments, K concentrations in lupin shoots significantly decreased when intercropped with wheat, whereas K concentrations in roots of intercropped lupin significantly decreased only at N3 (Fig. 2b, Table 3). S concentration was significantly reduced in lupin shoots and roots when intercropped with wheat, except for roots at N1 (Figs. 1b and 2b, Table 3). A significant effect of the intercropping on S concentration of wheat shoots was only observed at N3 (Fig. 2a, Table 3). In terms of Ca concentrations, the only significant differences were observed for wheat roots, whatever the N treatment (Fig. 2a, Table 3). For Mg concentrations, significant differences between crop treatments were only observed for wheat shoots at N2 and N3 (Fig. 2a), and for lupin shoots at N3 (Fig. 2b). Finally, like for K and S concentrations, Mg concentrations in shoots of intercropped wheat were significantly greater than those of sole wheat only at higher levels of soil mineral N.

## 2 Experimental Design, Materials and Methods

A calcareous cambisol was collected (0–15 cm depth) from the INRA experimental station located at Mauguio in Southern France (3°59′6″E, 43°37′13″N) three months after pea (*Pisum sativum* L.) harvest. The soil was sieved at 1-cm to remove any coarse organic material, air dried and stored in sealed buckets at ambient temperature until the establishment of the greenhouse experiment. Before greenhouse experiment, the soil was re-sieved with a 4 mm mesh and then mixed with perlite to allow better conditions for root development. Pots (4 dm^3^) were filled with 2 kg of soil and 0.2 kg of perlite. The final soil+perlite mixture exhibited the following mean properties: clay 202 g kg^−1^, silt 454 g kg^−1^, sand 343 g kg^−1^, total CaCO_3_ 39 g kg^−1^, pH_w_ 8.3, CEC_Metson_ 15 cmol_+_ kg^−1^, organic C 10.4 g kg^−1^, total N 0.95 g kg^−1^, ammonium (N—NH_4_^+^) 20 mg kg^−1^, nitrate (N—NO_3_^−^) 39 mg kg^−1^, inorganic available P (Olsen extraction method) 33.9 mg kg^−1^.

The greenhouse pot experiment was conducted at Center Mondial de l'Innovation (Roullier Group – Saint-Malo, France) during 61 days, using a full factorial design with three N treatments (N1 = 2, N2 = 33 and N3 = 65 mg N kg^−1^ dry soil added as a ^15^N-labelled urea) and three crop treatments: wheat (*Triticum aestivum* L. cv. Lennox) grown as sole crop; white lupin (*Lupinus albus* L. cv. Feodora) grown as sole crop; wheat-white lupin intercrop. In sole crop treatment, 12 seeds of wheat or 6 seeds of white lupin were sown per pot and thinned to 4 and 2 individuals, respectively, after emergence. For the intercrop treatment, seeds were sown at half the densities of those achieved for sole crops, *i.e*. 2 individuals of wheat and 1 individual of white lupin per pot. Pots were arranged in a complete randomized design with seven replicates. Throughout the course of the experiment, pots were automatically and daily watered to maintain water content at 75% of Water Holding Capacity (WHC = 28%) of the soil+perlite mixture. The air temperature in the greenhouse was 22.7 ± 2.1 °C, and the incident photosynthetically active radiation was 200 µmol s^−1^ m^−2^ on average, with a 16 h photoperiod.

Plants (shoots and roots) were harvested at 61 days after sowing, when most individuals of both species were in full flowering stage. For intercropped treatments, the root systems of the two species were carefully separated by hands to avoid excessive breakage. Within a pot, shoots or roots from all plants of a given species were assembled to constitute a unique shoot or root sample per species. Roots were carefully rinsed with deionized water in order to eliminate remaining soil particles. The dry weights of shoots and roots were measured after drying at 70 °C for 3 days. Potassium, Ca, Mg and S concentrations in shoots and roots were determined by Inductively Coupled Plasma Mass Spectrometry (ICP-MS) after digestion of 100 mg of finely ground material in a microwave oven (Multiwave PRO, Anton Paar, Austria) with concentrated HNO_3_ (65%) at 200 °C.

The effects of crop (intercropped or sole crop) and N treatments (N1, N2, N3) on K, Ca, Mg and S concentrations and accumulation in shoots and roots were initially tested by two-way ANOVA. In absence of interaction between factors (crop treatment x N treatment), one-way ANOVA was performed to test differences between crop treatments within a given N treatment. When necessary, data were squared root or log-transformed to cope with the ANOVA requirements. Significant differences between means were tested by Tukey's multiple comparison tests (*p* < 0.05). All statistical analyses were performed with R software v. 3.5.1 (R Core Team, 2018). The additional packages “car” and “agricolae” were used for ANOVA and Tukey's tests, respectively.

## CRediT authorship contribution statement

**Edith Le Cadre:** Conceptualization, Methodology, Writing – review & editing. **Ana Beatriz de Oliveira:** Conceptualization, Methodology, Writing – review & editing. **Mustapha Arkoun:** Writing – review & editing. **Jean Claude Yvin:** Writing – review & editing. **Philippe Hinsinger:** Conceptualization, Writing – review & editing.

## Declaration of Competing Interest

This research was funded by Center Mondial de l'Innovation (Roullier group) and supported by ANRT with a CIFRE agreement N° 2016/0875.

The authors declare that they have no known competing financial interests or personal relationships which have or could be perceived to have influenced the work reported in this article.
